# Correspondence

**Published:** 1895-07

**Authors:** Olof Schwarzkopf

**Affiliations:** McKillip Veterinary College, Chicago


### CORRESPONDENCE.

Editor Journal of Comparative Medicine and Veterinary Archives:

In your last issue you invite opinions in regard to a uniform degree in connection with Dr. Perry's letter on the subject. This gentleman has gallantly defended his own particular degree, but his arguments are not always correct. The "title" of V. S., as such, is not a misnomer, as he claims, because "surgeon" has been used in a general sense as signifying one who treats as well with medicine as with the knife. It is true, however, that "veterinary surgeon" is a pleonasm. The classical Latin "veterinarius" means one skilled in the healing of diseases of domestic animals, a surgeon and physician both. Grammatically, therefore, "veterinarian" is the most correct "title" which we can apply to ourselves. But we must distinguish between "title" and "degree." The English universities, from which our American institutions are largely copied, conferred the degrees of bachelor, master, and doctor. The last degree has originally been bestowed upon distinguished masters and teachers as an honorary token only. In America doctor degrees of all sorts have been given away to illiterate or half-educated men and women, and in consequence this degree has lost its original mark of distinction. We have arrived at a point where simplicity in titles means more than bluffness in degrees, hence the editor of this Journal was correct in recommending the simple V. S. as a title. Moreover, there is no country in the world which confers the degree of doctor on graduates of veterinary medicine but ours. The attaining of doctor degrees is a separate and distinct feature of university work. If we must have a doctor degree conferred by our American veterinary colleges, let us try to have one as near correct as possible. To my mind it matters little whether the degree is V.M.D., M.D.V., D.M.V., or D.V.S. One is just as poor Latin as the other, none is classical, but all of mediæval origin. It is true that the oldest degree in veterinary medicine, that of the University of Giessen, conferred first in 1796, reads: "Doctor medicine veterinariae." But besides this precedent we have nothing to go after, as far as I know. In thinking over the matter seriously the other day it appeared to me that "V.D," veterinariae doctor, would certainly be as good Latin as our American "M.D.," medicinae doctor. Moreover, it would doubtlessly be the most classical degree, although I have not asked counsel of a philologist. Whether this degree would suit the American instinct, I am curious to know; but it is short, decisive, and correct. As almost all of our American veterinary colleges have different degrees, all, with exception of one, would have to change the degrees, no matter which would be chosen, and if we adopt an entirely new degree no university or college could complain of having been wrongly treated.

In this connection I wish to call attention to the fact that we daily use bad pleonasm in our professional journals, evidently without realizing it. A "veterinary medical association" is something awfully wrong. There is our grand national association with the only title "United States Veterinary Medical Association." A foreigner, not familiar with the American branch of the English language, may break his jaw over this signature. Let us christen this worthy association with an appropriate name, say "American Veterinary Association," or something else correct. Let us begin here with our reform in presumptuous titles. OLOF SCHWARZKOPF.

McKILLIP VETERINARY COLLEGE, CHICAGO, MAY 5, 1895.

---

Illness has forced the retirement from active work of Prof. C. P. Lyman; we sincerely trust that it is of a very temporary character.

